# P031 Lost for words: reversible neurotoxicity in a patient with vasculitis

**DOI:** 10.1093/rap/rkag113.061

**Published:** 2026-11-03

**Authors:** Shreya Sanjaykumar Kalyani, Rashmikaa Netyam, Mariam Malik

**Affiliations:** Basingstoke and North Hampshire Hospitals NHS Trust, Basingstoke, United Kingdom; Basingstoke and North Hampshire Hospitals NHS Trust, Basingstoke, United Kingdom; Basingstoke and North Hampshire Hospitals NHS Trust, Basingstoke, United Kingdom

## Abstract

**Introduction:**

Methotrexate is a widely used disease-modifying agent in rheumatology, prescribed for various inflammatory conditions including giant cell arteritis (GCA). Although side effects of methotrexate are common, speech disturbance and neurotoxicity are rare with low-dose oral administration and are more typically seen in chemotherapeutic or intrathecal preparations, particularly in the paediatric population. This can pose a clinical dilemma, as symptoms may mimic a range of differentials, including cerebrovascular events.

**Case description:**

We present the case of an 84-year-old lady with a background of atrial fibrillation on anticoagulation, hypertension, and mild left ventricular dysfunction, who was diagnosed with GCA and new-onset polymyalgia rheumatica (PMR). GCA was confirmed on temporal artery biopsy. She was commenced on prednisolone, but due to recurrence of GCA symptoms during steroids tapering, methotrexate was introduced, and steroid weaning was continued. Six weeks into methotrexate treatment, she developed word-finding difficulties and gastric upset, with no clinical features of active GCA or PMR. A same-day emergency care review included a reassuring CT head; symptoms were attributed to oral candidiasis and steroid-related effects. Neurology and stroke referrals were made for further evaluation. Bloods showed CRP dropped from 74 at the time of diagnosis to 2 at the time of presentation with her new symptoms; ESR fell from 93 to 10. Haemoglobin was 118 g/L; WCC 9.2 × 10⁹/L; platelets 330 × 10⁹/L; eGFR 67mls/min; liver function, serum electrophoresis, and cholesterol levels were normal. Given her vasculitis history and recent speech disturbance, a CT angiogram was performed, which revealed near-complete contrast loss in the right vertebral artery and 50% stenosis at the left vertebral origin. The case was discussed at the Neuro-radiology MDT and findings were thought to be due to atheroma or chronic dissection. Vasculitis could not be ruled out, though clinical history was not supportive. MRI head showed mild small vessel disease. Her GP, while awaiting specialist review and investigations, temporarily withheld methotrexate to assess for drug-related side effects. At neurology specialist review, her symptoms had fully resolved. Imaging and test results were reassuring. The stroke specialist concluded that neither the symptoms nor imaging findings were consistent with stroke or active vasculitis. Her symptoms were felt to be due to methotrexate and resolved completely upon withdrawal.

**Discussion:**

This case highlights a rare but important adverse effect of methotrexate: transient speech disturbance suggestive of neurotoxicity. Although more commonly reported with high-dose or intrathecal administration in oncology settings, this case demonstrates that neurotoxic effects can also occur with doses used in rheumatology. Given the broad differential for acute speech disturbance—including cerebrovascular events and disease relapse—methotrexate-induced neurotoxicity should be considered, particularly when other investigations are unremarkable and symptoms resolve upon drug cessation. Early recognition can prevent unnecessary investigations, support safer prescribing, and guide appropriate drug monitoring in vulnerable populations.

**Key learning points:**

1. Methotrexate related neurotoxicity can occur even with low-dose oral therapy.

2. Speech disturbance may be an isolated manifestation of methotrexate toxicity.

3. Clinical assessment, inflammatory markers, and imaging abnormalities should be interpreted in the clinical context.

4. Drug withdrawal can be both diagnostic and therapeutic in some contexts such as these if it is safe to do so.

5. Multidisciplinary collaboration improves diagnostic accuracy - the neurologist’s assessment in this patient helped with pinning the diagnoses down with diagnostic confidence and lead to favourable outcomes.

